# No association between alexithymia and emotion recognition or theory of mind in a sample of adolescents enhanced for autistic traits

**DOI:** 10.1177/13623613231221928

**Published:** 2024-01-19

**Authors:** Georgianna Moraitopoulou, Hannah Pickard, Emily Simonoff, Andrew Pickles, Rachael Bedford, Virginia Carter Leno

**Affiliations:** 1 Social Genetic and Developmental Psychiatry Centre, Institute of Psychiatry, Psychology and Neuroscience (IoPPN), King’s College London, London, UK; 2 Department of Child and Adolescent Psychiatry, Institute of Psychiatry, Psychology and Neuroscience (IoPPN), King’s College London, London, UK; 3 Department of Psychological Sciences, Birkbeck, University of London, London, UK; 4 Department of Biostatistics and Health Informatics, Institute of Psychiatry, Psychology and Neuroscience (IoPPN), King’s College London, London, UK; 5 Department of Psychology, University of Bath, Bath, UK

**Keywords:** adolescence, alexithymia, autism, emotion recognition, theory of mind

## Abstract

The alexithymia hypothesis posits that specific aspects of the autistic socio-cognitive profile, namely emotion recognition difficulties, can be explained by the increased prevalence of alexithymia in autistic populations. However, this hypothesis has largely been tested in adults. We tested whether co-occurring alexithymia could account for associations between autistic traits and two key domains of socio-emotional cognitive functioning in adolescence. Participants were 184 (44% male) children aged 10–16 years with and without a diagnosis of autism (n = 75 autistic), who completed experimental measures of emotion recognition and theory of mind. Parents completed assessment of autistic traits and alexithymia. Pre-registered analyses tested associations between autistic traits and socio-cognitive task performance, and whether including alexithymia as an additional predictor changed the nature of the association between autistic traits and task performance. Results showed participants with higher levels of autistic traits performed lower on emotion recognition and theory of mind tasks, and that these associations were not significantly altered by including alexithymia in the models. Results suggest that more work is needed to understand the applicability of the alexithymia hypothesis in younger populations, but that at least in young adolescents and when using parent-report measures, alexithymia may not be associated with emotion recognition or theory of mind ability.

**Lay abstract:**

Alexithymia is a sub-clinical condition characterised by difficulties identifying and describing one’s own emotions, which is found in many, but not all autistic people. The alexithymia hypothesis suggests that certain aspects of socio-cognitive functioning typically attributed to autism, namely difficulties in emotion recognition, might be better explained by often co-occurring alexithymia. It is important to understand what is specific to autism and what is due to other co-occurring characteristics to develop appropriate support for autistic people. However, most research on this topic has been conducted in adults, which limits our knowledge about the relevance of this theory to younger autistic populations. This study tested whether difficulties in emotion recognition and theory of mind traditionally associated with autism might be better explained by alexithymia in a sample of adolescents with and without a diagnosis of autism. Results found that difficulties in emotion recognition and theory of mind were both associated with autistic traits, and this was not accounted for by individual differences in levels of alexithymia. This research suggests that more work is needed to understand the applicability of the alexithymia hypothesis in younger populations, but that at least in adolescents and when using parent-report measures, alexithymia may not account for emotion recognition or theory of mind difficulties associated with autistic traits.

## Introduction

Autistic youth often experience difficulties in social interaction and communication, which are thought to be in part driven by difficulties in aspects of socio-cognitive functioning. However, growing awareness of the overlap between autism and alexithymia (Kinnaird et al., 2020), a sub-clinical condition defined by difficulties in identifying and describing one’s own emotions (Samur et al., 2013), and reports that alexithymia is associated with socio-emotional difficulties typically attributed to autism, has raised questions as to whether certain aspects of the autistic cognitive profile could be better explained by co-occurring alexithymia (Bird & Cook, 2013). Most research in the field has focused on adults, despite adolescence being a key period in developing socio-cognitive abilities (Blakemore, 2012; Lawrence et al., 2015; Thomas et al., 2007), and meta-analyses suggesting differences in emotional self-awareness (conceptually similar to alexithymia) between autistic and non-autistic people begin to emerge during adolescence (Huggins et al., 2021). Exploring whether comparable effects are found in adolescence is crucial to understanding the developmental specificity of the proposed role of alexithymia in the autistic cognitive profile.

Alexithymia is a sub-clinical condition characterised by difficulties identifying and describing one’s own emotions and an externally orientated cognitive style (Taylor & Bagby, 2000). Alexithymia frequently co-occurs with autism (Kinnaird et al., 2020), among other conditions (e.g. anxiety, depression, eating disorders; Berthoz et al., 1999; Rozenstein et al., 2011), and is associated with difficulties in emotion recognition (Grynberg et al., 2012), consistent with the idea that recognition of others’ emotions in part relies on one’s own subjective experience of emotion (Sato et al., 2013). Bird and Cook (2013) proposed the *alexithymia hypothesis* that emotional difficulties attributed to autism may be better explained by co-occurring alexithymia. This hypothesis is supported by reports that associations between emotion recognition and autism in adults are no longer present when analyses adjusted for individual differences in alexithymia (similar effects have also been found for somatoform disorder and eating disorders; Brewer et al., 2015; Pedrosa Gil et al., 2009). Similarly, alexithymia is found to be a better predictor than autism of emotion recognition (Cook et al., 2013; Ola & Gullon-Scott, 2020), empathic responsivity as indexed by neural activation (Bird et al., 2010) and patterns of gaze during viewing of emotional facial expressions (Cuve et al., 2021). However, all the aforementioned studies were conducted in adult populations. In one of the few studies that examined the impact of alexithymia in adolescence, alexithymia was associated with difficulties in the recognition of angry faces in autistic adolescents, but covariation analyses suggested this was largely due to the overlap of alexithymia and verbal IQ (VIQ; Milosavljevic et al., 2016).

In addition to accurate recognition of emotions, another key socio-cognitive ability that is often implicated in autism is theory of mind (ToM); the ability to understand and predict others’ behaviour in terms of their internal mental states. Across different types of ToM tasks, studies find that autistic youth score lower than their typically developing peers (Brunsdon et al., 2015; Burnside et al., 2017; Wilson, 2021) (although see Crompton et al., 2020; Milton, 2012, for a critique of ToM models more broadly). Conceptually, ToM is separable but related to emotion recognition; reasoning why someone experiences certain emotions (a core part of ToM) in part relies on accurate identification of emotional cues (Bird & Viding, 2014). This opens up questions as to whether alexithymia could also impact on ToM abilities (via its impact on emotion recognition). Currently, the association between alexithymia and ToM is less well investigated, with some reporting that alexithymia is associated with lower ToM task performance (Al Aïn et al., 2013; Lombardo et al., 2007; Moriguchi et al., 2006) while others report null findings (Lockwood et al., 2013), or associations between alexithymia and ToM are no longer significant once adjusting for IQ, age and level of autistic traits (Gökçen et al., 2016). One study found that autistic adults with high levels of alexithymia scored lower on a self-report measure of cognitive empathy (conceptually similar to ToM abilities) than both typically developing and autistic individuals without alexithymia (Mul et al., 2018). The fact that the group of autistic individuals without alexithymia still scored lower than the typically developing group suggests that both alexithymia and autism contribute to ToM-type difficulties. Other studies using self-report measures also find both autistic traits and alexithymia are associated with lower cognitive empathy (Shah et al., 2019). A recent systematic review of ToM, and meta-analysis where sufficient data were available, suggests that the inconsistency in findings could be in part explained by contamination of certain ToM tasks (e.g. Reading the Mind in the Eyes task (Baron-Cohen et al., 2001) with emotion recognition abilities (Pisani et al., 2021). The authors suggest that if alexithymia is associated with difficulties in emotion recognition, ToM measures that require accurate emotion recognition to infer accurate mental states may be negatively associated with alexithymia purely because of their reliance on accurate emotion recognition. Indeed, studies which administer measures of ToM that are less reliant on emotion recognition abilities find alexithymia is not associated with ToM when accounting for individual differences in autism traits (Oakley et al., 2016). Overall, the meta-analysis by Pisani and colleagues suggested no reliable association between alexithymia and ToM on measures that do not involve emotional ability; however, only one of the included studies was conducted with adolescents. In this study of a relatively modest sample of predominantly male autistic adolescents, alexithymia was not associated with ToM performance (Milosavljevic et al., 2016). Since the systematic review by Pisani and colleagues was published, one additional study has been conducted in a sample of largely neurotypical youth aged 8–12 years, with analyses finding alexithymia was a more consistent predictor than autistic traits of lower scores on self and parent-reported empathy measures, which in part tap ToM abilities (Speyer et al., 2022). However, these empathy measures likely index a range of socio-cognitive processes beyond core ToM abilities, for example, emotion recognition and affective resonance. There is a lack of research with specific and objective measures of ToM in adolescence, despite a wealth of literature suggesting that adolescence is a sensitive period for socio-cognitive development, with studies in typically developing populations finding adolescence is characterised by ongoing improvements in emotion recognition (Thomas et al., 2007) and ToM abilities (Dumontheil et al., 2010), and increased activity in cortical areas involved in social cognition during ToM tasks (Kilford et al., 2016).

The present paper extends existing adult research by testing associations between autistic traits, alexithymia, emotion recognition, and ToM in adolescence. We have previously shown in this sample that when adjusting for callous unemotional traits, higher autistic traits are related to lower emotion recognition accuracy when cued to the eyes (Carter Leno et al., 2022). Here, we test a different set of hypotheses, for some of which we use data from the same emotion recognition task. Following our pre-registered hypotheses (https://osf.io/pw65j), we predict that in addition to autistic traits, alexithymia will also be associated with lower emotion recognition accuracy; however, the strength of the association between autistic traits and emotion recognition will be significantly reduced once alexithymia is accounted for (in line with the alexithymia hypothesis). We also predict that higher autistic traits will be associated with lower ToM performance; however, due to insufficient literature, we did not have specific hypotheses as to the directionality of the ToM and alexithymia effect, nor whether alexithymia could account for the association between autistic traits and ToM. We use a ToM task that has minimal emotional cues to minimise contamination with emotion recognition difficulties.

## Method

### Participants

Participants were recruited via secondary schools, charities and social media. An additional targeted recruitment drive aimed at increasing variability in autistic traits in the sample recruited through schools specifically for children with social, emotional and behavioural difficulties. Inclusion criteria were being 10–16 years of age, living in the United Kingdom and being fluent in English. Exclusion criteria were the child having parent-reported genetic or psychotic conditions. Autism diagnosis was recorded using parent-reported diagnostic information (parents were asked which diagnostic label, who gave the diagnosis and at which age the diagnosis was received, see Supplemental Table S1). Parent questionnaire data was collected online using Qualtrics and child cognitive task data was collected online using Gorilla (https://gorilla.sc). Where two siblings took part (n = 2), data from one sibling was excluded at random. Full sample characteristics are reported in Table 1. To be included in the current analysis, participants had to have complete measurement of emotion recognition, sex, age, autistic traits and alexithymia, giving a final sample of N = 184. Exclusions were as follows: N = 204 participants completed the emotion recognition task, N = 203 participants had valid emotion recognition task data and N = 184 participants had valid emotion recognition data and valid covariate data (see below for details of task-level exclusions). We tested for group differences in age, sex, ethnicity (coded as White vs non-White for statistical analysis), parental education, VIQ, Confusion, Hubbub and Order Scale (CHAOS) total, and autistic traits between the final analysis sample who had valid data on the emotion recognition task and all primary model covariates (n = 184) and the subsample who had complete data on the first part of the emotion recognition task only and were therefore excluded from the analysis (n = 19). Fischer’s exact test was used for binary variables (sex, ethnicity) and t-tests for continuous variables. All comparisons were non-significant (ps > 0.09). All parents provided online consent for themselves and their child to take part and children provided online assent. This study was approved by the Psychiatry, Nursing and Midwifery Research Ethics Committee, King’s College London (HR-19/20-17193) and Bath Ethics Committee (Psychology Research Ethics Committee reference number 20-199).

**Table 1.** Sample descriptives.

Variables mean (SD; range)	N	Whole sample	Autistic (n = 75)	Non-autistic (n = 109)	Non-autistic with SCQ < 15 (n = 88)
Males:females (% male)	184	81:103 (44%)	39:36 (52%)	42:67 (39%)	32:55 (36%)
Age (years)	184	13.09 (1.76; 10–16)	13.18 (1.80)	13.04 (1.74)	13.00 (1.66)
Parental education <UG degree: ⩾UG degree (% ⩾UG degree)	184	32:148 (82%)	11:64 (85%)	21:84 (80%)	12:74 (86%)
Ethnicity					
White English/Welsh/Scottish/Northern Irish/British	184	144 (78%)	62 (83%)	82 (75%)	60 (74%)
Other White background		15 (8%)			
Other mixed/multiple background		7 (4%)			
Indian		3 (2%)			
Other Ethnic group		3 (2%)			
Black/African/Caribbean/Black British background		3 (2%)			
African		2 (1%)			
Other categories with <n = 2 participants		7 (4%)			
CHAOS score	182	4.41 (3.75; 0–14)	4.87 (3.94)	4.09 (3.60)	3.42 (3.25)
SCQ-Lifetime	182	12.99 (9.21; 0–32)	20.04 (6.25)	8.05 (7.60)	4.79 (3.74)
AQ-10	184	5.75 (3.00; 0–10)	8.08 (1.59)	4.15 (2.67)	3.41 (2.32)
CAM-PR	184	15.46 (11.24; 0–42)	20.67 (10.64)	11.88 (10.23)	8.90 (6.75)
Emotion recognition accuracy	184	79.05 (12.01; 38.33–100)	75.20 (13.00)	81.70 (10.55)	82.82 (9.92)
ToM Intentionality	163	3.01 (0.90; 0–4.75)	2.82 (0.95)	3.16 (0.84)	3.25 (0.81)
ToM Accuracy	163	0.85 (0.49; 0–2)	.74 (0.48)	0.94 (0.49)	0.97 (0.50)
VIQ standardised score	177	119.56 (18.87; 55–145)	115.38 (19.92)	122.43 (17.64)	125.96 (14.48)

SD: standard deviation; SCQ: Social Communication Questionnaire; UG: undergraduate; CHAOS: Confusion, Hubbub and Order Scale; AQ: Autism Quotient–Adolescent version; CAM-PR: Children’s Alexithymia Measure–Parent Report; ToM: theory of mind; VIQ: verbal IQ.

Participants in the non-autistic group were excluded from sensitivity analyses if they had a total SCQ-lifetime score of ⩾15.

### Experimental measures

#### Emotion recognition task

As reported previously (Carter Leno et al., 2022), participants were shown a block of 20 trials consisting of faces expressing 5 emotions (happiness, sadness, surprise, anger, fear), with 4 trials per emotion. Four different face prototypes were used, taken from Griffiths et al. (2019) (see Griffiths et al., 2015, for more detail), including two European faces (one male and one female) and two South Asian faces (one male and one female). An intensity level of 6 (from a possible range of 0–8) was selected to ensure sufficient variability in response accuracy (based on the location of maximal group differences in Griffiths et al., 2019). Each trial consisted of a central fixation cross on the screen for 1000 ms, followed by the face stimulus for 2000 ms, followed by five emotion labels presented on the screen. The position of the emotion labels was randomly chosen for each participant but remained consistent throughout all remaining trials. Participants were instructed to use the cursor to choose the emotion label that they thought best represented the emotion of the face. Overall accuracy was computed as a percentage (i.e. sum of correct responses/total number of valid trials × 100). Trials with reaction times (RTs) below 200 ms or 3 standard deviations (SDs) above each participant’s mean task RT (2.4% of trials), or where Gorilla noted a stimulus loading delay (0.3% of trials) were excluded. At the task level, data were excluded if (a) the participant noted that the stimuli did not present properly (n = 0), (b) loading delays were present on ⩾20% of trials (n = 0), (c) the stimulus did not render at the correct size (n = 1) or (d) participants had <50% valid trials (n = 0). Following these exclusions, one participant was excluded, and the remaining participants had an average of 97% valid trials.

#### Frith-Happé Animations Task

The Frith-Happé Animations Task (Abell et al., 2000) was used to capture a measure of ToM intentionality and accuracy. Participants were shown five animations of triangles interacting: one goal-directed (fighting) and four mental state interactions (surprising, seducing, mocking and coaxing). Each trial consisted of a fixation cross on the screen for 1000 ms, followed by the video cartoon animation. Once the video had finished, participants were presented with an open text box and instructed to type ‘what was happening in the cartoon?’. Participants were always presented with the goal-directed animation (fighting) first, followed by the mental state animations presented in a random order. For each animation, the typed descriptions were coded for accuracy (0–2) and intentionality (0–5), measuring the level of understanding of the events and the level of mental state descriptions used for each animation, respectively. All transcripts were coded by a single researcher (G.M.), blind to diagnostic status. Thirty transcripts were double coded (by H.P.), and inter-rater reliability was good (α = 0.85). Average accuracy and intentionality were calculated for the goal-directed and mental state conditions separately, with our primary metric of interest being average scores in the mental state condition. Recent work confirms that online administration of this task operates in a comparable manner to in-person administration (Livingston et al., 2021). Data from the ToM task were excluded if the participant had incomplete/inconclusive data on >2 mental state animations (n = 2).

#### Receptive One Word Picture Vocabulary Test – 4th edition

The Receptive One Word Picture Vocabulary Test – 4th edition (ROWPVT-4) is a vocabulary test designed for individuals from 2 to 80+ years, adapted for online use following approval from the publishers (Brownell, 1987). On each trial, the participant was presented with four pictures and an audio clip of a word that matches one of the four pictures. Participants were given the option to replay the audio clip as many times as required and select the picture that matches the word. Verbal IQ (VIQ) was calculated by summing the correct responses across the 190 experimental trials and converting the raw total to standardised scores. Trials with RTs < 200 ms (0.2% of trials) or where Gorilla noted visual or auditory stimuli had not properly loaded were excluded (<0.1% of trials). Data were excluded overall if the participant noted that the stimuli did not present properly on the task (n = 2) and if loading delays were present on ⩾20% of trials (n = 0).

### Parent/caregiver questionnaires

#### Confusion, Hubbub and Order Scale

CHAOS (Matheny et al., 1995) is a 15-item parent-report questionnaire used to measure environmental factors in the household, with higher scores indicating a noisier, more distracting and stressful home environment. This measure was used to control for the potential impact of background home environment on task performance. The CHAOS has been reported to show high concurrent validity with direct measures of the physical and social environment (Matheny et al., 1995). In the current study, internal consistency was good (Cronbach’s α = 0.85).

#### Social Communication Questionnaire–Lifetime version

The Social Communication Questionnaire–Lifetime version (SCQ-L; Rutter et al., 2003) is 40-item parent-report questionnaire, where parents and caregivers are asked ‘yes’ or ‘no’ questions to assess lifetime autistic traits. Scores ⩾15 are suggestive of the presence of autism. The SCQ-L was included as a screener to detect potentially missed autism diagnoses for children in the non-autistic group.

#### Autism Quotient – 10-item Adolescent version

A modified version of the original self-report Autism Quotient (AQ) (Baron-Cohen et al., 2006) for adolescents was used to measure autistic traits in the sample (AQ-10; Allison et al., 2012). Parents were asked to rate 10 statements from ‘definitely agree’ to ‘definitely disagree’. This shorter version was adapted from the longer 50-item version by selecting the 10 most discriminating items, based on a discrimination index (calculated by subtracting the proportion of participants who scored 1 on each item in the control group from the proportion of participants who scored 1 in the autistic group), in a sample of autistic (N = 162) and typically developing control (N = 475) adolescents aged 12–15 years. Cronbach’s alpha for the AQ-10 Adolescent version was 0.89, and scores on the 10-item version were significantly correlated with the original AQ-50 Adolescent version (r = 0.95, p < 0.0001). In the current study, internal consistency was good (Cronbach’s α = 0.82).

#### Children’s Alexithymia Measure–Parent Report

The Children’s Alexithymia Measure–Parent Report (CAM-PR) (Way et al., 2010) is a 14-item questionnaire designed to measure alexithymia in children aged 5–17 years. Parents were asked to report on their child’s behaviour over the past 3 months, by rating the applicability of statements on a 4-point Likert-type scale from 0 (‘almost never’) to 3 (‘almost always’), with higher scores indicating higher alexithymia. The CAM-PR had excellent internal consistency in the current sample (Cronbach’s α = 0.95), similar to that in the original validation study (Cronbach’s α = 0.92 in Way et al., 2010; N = 220 in 5–17 year olds). Others have reported good reliability in samples of typically developing children and children with developmental language disorder aged 9–16 years (Cronbach’s α = 0.91 for both; Hobson & van den Bedem, 2021).

#### Additional exclusion criteria

To ensure data quality, attention checks were presented twice throughout the battery of tasks. The attention check consisted of six animal pictures and participants were asked, for example, to ‘please click on the fish’. All participants passed this criterion. Our final sample size used in the analysis was N = 184 for the emotion recognition task and N = 163 for the ToM task.

### Statistical analysis

The data analysis plan was pre-registered (https://osf.io/pw65j). Data cleaning was conducted in R and analyses were conducted in Stata 16. Summary statistics were calculated on the complete sample and additionally for the autistic and non-autistic samples to aid sample characterisation. Correlations between all variables are presented in Supplemental Table S2. Examination of the variance inflation factor (VIF) for autistic traits and alexithymia suggested that multi-collinearity was not great enough to prevent us entering both predictors in the same model (VIF = 1.79). Associations between autistic traits, alexithymia and emotion recognition were tested using linear regression, with emotion recognition entered as the dependent variable. For ToM, we used a seemingly unrelated regression model as this approach allowed us to test predictors of correlated dependent variables (intentionality and accuracy) in one model. In primary models, age, sex and autistic traits were included as covariates (Step 1) and then alexithymia was added to assess whether it was associated with variability in cognitive performance over and above autistic traits (Step 2). In secondary analyses, we re-ran all models including parental education (a proxy for socio-economic status [SES]), household CHAOS and VIQ to assess the impact of these factors on results (see Supplementary Materials). All models were estimated with robust standard errors to account for heteroskedasticity. We present both unstandardised (b) and standardised (β) coefficients. We also tested the statistical significance of the change in the association between autistic traits and socio-cognitive task performance when alexithymia was included by calculating the difference in beta coefficients and dividing this difference by the standard error of the difference. This gives a t statistic which can be combined with the degrees of freedom to find the associated p value (Clogg et al., 1995). Although not specified in our original analysis plan, to aid interpretation of effects beyond p values, we also calculated the Bayes factor (BF_10_) for the alexithymia term compared against a model with age, sex and autistic traits using JASP (10.31234/osf.io/pqju6), with interpretation of values based on published thresholds (Wagenmakers et al., 2018).

We deviated from our pre-specified analyses by (1) not including diagnostic group as this complicated the interpretation of effects (e.g. this would be testing the association with autistic traits while adjusting for diagnostic status). Instead, as a follow-up analysis, we tested whether associations between alexithymia and social cognition were different in the autistic group compared with the non-autistic group, in part to understand if any null effects could be driven by a narrower distribution of alexithymia in the non-autistic group. This was done by including an alexithymia × group term as a predictor. Models including the interaction term excluded youth in the non-autistic group above threshold on the SCQ-L (⩾15; n = 16) to ensure potential autistic adolescents in the non-autistic group (who did not have a clinical diagnosis) were not mis-classified. Due to the heterogeneity of autism (i.e. some autistic individuals show greater social difficulties than others), no autistic adolescent was removed due to scoring below the SCQ-L cut-off in our primary analyses (although we include supplementary analyses where we exclude these 12 participants for completeness, see Supplemental Tables S6–S9). We also deviated by (2) entering autistic traits and then autistic traits + alexithymia as predictors in primary models (i.e. not running a model with alexithymia without autistic traits, again due to group differences in the distribution of alexithymia); and (3) for parsimony, combining VIQ into the secondary covariate models (rather than running a third set of covariate models, as laid out in our pre-registration).

## Community involvement

Community members were not involved in this study.

## Results

See Table 1 for sample descriptives.

### Associations between emotion recognition, autistic traits and alexithymia

In Step 1, regression models showed autistic traits were significantly associated with emotion recognition ability (b = −0.73, 95% confidence interval (CI [−1.33 to −0.12], p = 0.02, β = −0.18). Sex was also significantly associated with emotion recognition ability (b = 3.98, 95% CI [0.42 to 7.54], p = 0.03, β = 0.17), with females scoring higher than males, while age was not significantly associated with emotion recognition ability (b = −0.01, 95% CI [−1.13 to 1.12], p = 0.99, β = −0.01). In Step 2, when alexithymia was included in the model (see Table 2), the association with autistic traits became statistically non-significant (b = −0.73, 95% CI [−1.51 to 0.04], p = 0.06, β = −0.18), and alexithymia was not associated with emotion recognition performance (b = 0.01, 95% CI [−0.19, 0.19], p = 0.98, β = 0.01). Including alexithymia did not significantly modulate the strength of the association between autistic traits and emotion recognition performance (t = 0.60, p = 0.55), and BF_10_ = 0.294 suggests moderate evidence for the null (i.e. no effect of alexithymia). In secondary models including parental education, CHAOS total and VIQ, the pattern of results was not substantially changed (see Supplemental Table S3 for full output), although autistic traits were no longer significantly associated with emotion recognition (b = −0.59, 95% CI [−1.26 to 0.07], p = 0.08, β = −0.15). In additional moderation analyses, the group × alexithymia interaction term was not a significant predictor of emotion recognition task performance (b = −0.23, 95% CI [−0.63 to 0.17], p = 0.25).

**Table 2.** Primary models testing associations between autistic traits, alexithymia and emotion recognition task performance.

Predictor	b	β	95% confidence intervals		p value
			Lower bound	Upper bound	
Age (years)	−0.01	−0.01	−1.13	1.12	0.99
Sex	3.98	0.17	0.42	7.54	**0.03**
Autistic traits	−0.73	−0.18	−1.33	−0.12	**0.02**
Age (years)	−0.01	−0.01	−1.13	1.13	0.99
Sex	3.98	0.17	0.41	7.55	**0.03**
Autistic traits	−0.73	−0.18	−1.51	0.04	0.06
Alexithymia	0.01	0.01	−0.19	0.19	0.98

Bold values indicate *p* < 0.05.

### Associations between ToM, autistic traits and alexithymia

In Step 1, regression models showed higher autistic traits were significantly associated with lower ToM intentionality scores (b = −0.06, 95% CI [−0.10 to −0.01], p = 0.01, β = −0.19). Age was positively associated with intentionality (b = 0.14, 95% CI [0.07 to 0.21], p < 0.01, β = 0.26). Sex was not significantly associated with intentionality (b = 0.21, 95% CI [−0.05 to 0.48], p = 0.11, β = 0.11). In Step 2, when alexithymia was included in the model, the pattern of associations did not change (see Table 3), such that higher autistic traits remained associated with lower intentionality scores (b = −0.07, 95% CI [−0.13 to −0.01], p = 0.03, β = −0.21), and alexithymia was not associated with intentionality scores (b = −0.01, 95% CI [−0.01 to 0.02], p = 0.59, β = 0.05). Including alexithymia did not significantly change the strength of the association between autistic traits and intentionality (t = 0.45, p = 0.65), and BF_10_ = 0.314 suggested moderate evidence for the null. A comparable pattern of associations was found with ToM accuracy, and as before, including alexithymia did not significantly change the strength of the significant association between autistic traits and accuracy (t = 0.89, p = 0.37), and BF_10_ = 0.393 suggests weak evidence for the null. As a final sensitivity analysis, we re-ran primary models with Goal-Directed scores (with accuracy scores of 1 recoded to 0) included as covariates to check that differences in task understanding were not contributing to the pattern of results. Results remained unchanged (see Supplemental Table S4 for full output).

**Table 3.** Primary models testing associations between autistic traits, alexithymia and ToM task performance.

	b	β	95% confidence intervals		p value
			Lower bound	Upper bound	
ToM intentionality					
Age (years)	0.14	0.26	0.07	0.21	**<0.01**
Sex	0.21	0.11	−0.05	0.48	0.11
Autistic traits	−0.06	−0.19	−0.10	−0.01	**0.01**
Age (years)	0.13	0.26	0.07	0.21	**<0.01**
Sex	0.21	0.12	−0.06	0.48	0.11
Autistic traits	−0.07	−0.21	−0.13	−0.01	**0.03**
Alexithymia	<0.01	0.05	−0.01	0.02	0.59
ToM accuracy					
Age (years)	0.08	0.27	0.04	0.12	**<0.01**
Sex	0.09	0.09	−0.05	0.23	0.21
Autistic traits	−0.04	−0.22	−0.06	−0.01	**<0.01**
Age (years)	0.08	0.27	0.04	0.12	**<0.01**
Sex	0.09	0.09	−0.05	0.23	0.22
Autistic traits	−0.05	−0.28	−0.08	−0.01	**<0.01**
Alexithymia	<0.01	0.09	−0.00	0.01	0.35

ToM: theory of mind.

Bold values indicate *p* < 0.05.

In secondary models including parental education, CHAOS total and VIQ as covariates, the association between autistic traits and intentionality fell below significance (b = −0.03, 95% CI [−0.07 to 0.02], p = 0.26, β = −0.09), but the association with accuracy remained (b = −0.03, 95% CI [−0.05 to −0.01], p = 0.03, β = −0.16). As before, alexithymia was not significantly associated with intentionality or accuracy (b = 0.01, 95% CI [0.01 to 0.02], p = 0.42, β = 0.08; b = 0.01, 95% CI [−0.01 to 0.01], p = 0.20, β = 0.12) (see Supplementary Table 5 for full output).

**Figure 1.**
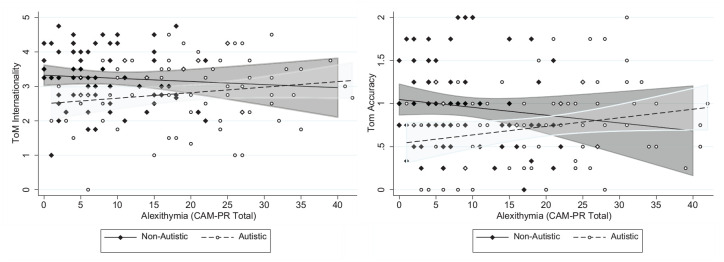
The direction of alexithymia-theory of mind (ToM) associations differs between autistic and non-autistic adolescents.

In additional moderation analyses, the group × alexithymia interaction was non-significant for intentionality (b = 0.03, 95% CI [−0.01 to 0.06], p = 0.06), but was significant for accuracy (b = 0.02, 95% CI [0.01 to 0.04], p = 0.01), and remained at significance when adjusting for parental education, CHAOS total and VIQ (b = 0.02, 95% CI [−0.001 to 0.03], p = 0.05). Running models in autistic and non-autistic groups separately showed a negative association between alexithymia and accuracy in the non-autistic group (b = −0.02, 95% CI [−0.03 to −0.01], p = 0.03, β = −0.21), and some weak evidence for a positive association in the autistic group (b = 0.01, 95% CI [−0.01 to 0.02], p = 0.08, β = 0.19) (see Figure 1). Associations in both the non-autistic and autistic groups separately fell below significance when adjusting for parental education, CHAOS total and VIQ (p = 0.35 and 0.13, respectively).

## Discussion

This study tested the relevance of the *alexithymia hypothesis* to adolescent populations. Understanding what is specific to autism instead of driven by co-occurring characteristics is key to understanding the socio-cognitive profile associated with autism. Results showed autistic traits were associated with lower emotion recognition and lower ToM task performance. There was no significant association between emotion recognition and alexithymia in models including age, sex and autistic traits as predictors. Similarly, we found no association between alexithymia and ToM, and our follow-up moderation analyses suggested that the lack of association between alexithymia and social cognition was not due to reduced variability of alexithymia scores in the non-autistic group.

Based on the alexithymia hypothesis, we predicted that the association between autistic traits and emotion recognition would be fully accounted for by alexithymia, such that the association would become non-significant in models that included alexithymia as an independent variable, and the coefficient of effect for autistic traits would be significantly lower than in models without alexithymia. Although the association between autistic traits and emotion recognition did become non-significant when alexithymia was included (shifting to p = 0.06), the standardised coefficient of effect did not change, and the change in effect was statistically non-significant. Alexithymia was not itself significantly associated with emotion recognition performance. The calculation of Bayes factors for the association with alexithymia indicated weak to moderate evidence for the null hypothesis – that is, there is no effect of alexithymia on socio-cognitive task performance over and above age, sex and autistic traits. We note that in secondary covariate models where we adjusted for parent education, background environment and verbal IQ, the association between autistic traits and emotion recognition was non-significant (p = 0.08), although the standardised coefficient of effect was similar between primary and secondary models (β = 0.18 vs β = 0.15). This change in effect may in part represent reduced precision with the addition of more covariates, but we have found in this sample that when adjusting for other characteristics that impact emotion recognition (e.g. callous-unemotional traits), autistic traits are only associated with emotion recognition difficulties when specifically cued to the eyes (unlike here, where we did not cue eye gaze; Carter Leno et al., 2022).

Although a central tenant of frameworks that seek to explain alexithymia is that difficulties differentiating emotions in the self leads to impaired understanding of emotions in others (Bird & Viding, 2014), current results suggest that at least in adolescence, this may not be the case. Indeed, most existing reports of alexithymia correlating with, and accounting for emotion recognition difficulties, have largely been conducted in adult populations (Cook et al., 2013; Ola & Gullon-Scott, 2020). In other adolescent populations, similar null results are reported elsewhere, with one study of autistic participants finding no association between self-reported alexithymia and emotion recognition when adjusting for VIQ (Milosavljevic et al., 2016).

There are several reasons that could explain the discrepancy between adult studies that find associations between emotion recognition and alexithymia and null findings in adolescent samples. Although alexithymia is often described as a trait, which implies stability over time, one explanation is that alexithymia is still emerging throughout adolescence (indeed although non-significant, we note a positive correlation between age and alexithymia in the current sample, r = 0.14, p = 0.07). This could mean that the effects of alexithymia on social cognition are not apparent until late adolescence/adulthood. This would be in line with reports that emotion recognition abilities are still developing between adolescence and adulthood (Rodger et al., 2015). Thus, it may have been that our sample’s age range of 10–16 years was too young to capture the children with delays in developing of emotion recognition skills – and these may be the children who also have higher levels of alexithymia in late adolescence/early adulthood. This hypothesis of emerging difficulties is in line with a meta-analysis that found group differences in alexithymia between autistic and typically developing people are not present in childhood, emerging in adolescence and clearest in adulthood (Huggins et al., 2021).

An alternative explanation for the current null results with regard to testing the alexithymia hypothesis is our use of a parent-rated measure to capture levels of alexithymia, unlike in adult studies, where self-report measures are typically used. Although the validity of self-report measures of alexithymia has been established in autistic adults (Berthoz & Hill, 2005; Samson et al., 2012) (however, see Williams & Gotham, 2021, who suggest limitations of particular measures), to our knowledge, no study has examined the psychometric properties of self-report measures of alexithymia in autistic children and adolescents, or demonstrated acceptable reliability in typically developing children with similar ages to our lowest age bound (10 years). Indeed, the most widely used self-report measure of alexithymia (e.g. the TAS-20 [Toronto Alexithymia Scale – 20]) has been found to have lower, and at times unacceptable, reliability on multiple subscales in non-autistic children and adolescents (Parker et al., 2010; Säkkinen et al., 2007). Others have re-worded the TAS to have more appropriate language for children; however, one of the three subscales (Externally Oriented Thinking) was still found to have unacceptable reliability in primary and secondary school children (Cronbach’s alpha = 0.29) (Rieffe et al., 2006). Therefore, we chose to use a parent-report measure in the current study because we felt that self-report may not be suitable for the younger participants in our sample of 10–16 year olds, and especially those who were also autistic or had high levels of autistic traits, who may have additional difficulties identifying and communicating their internal states. Given that correlations between self and parent-report measures of alexithymia in neurotypical and autistic youth (Griffin et al., 2016; Lampi et al., 2021; Speyer et al., 2022) and children with developmental language disorder (Hobson & van den Bedem, 2021) are generally not significant (although see Brown et al., 2022, for an exception with a recently developed measure), parent versus self-report measures may be measuring different things, and this could in part explain why our results differ from those reported from adult studies that used self-report. Indeed, a recent review highlighted that the type of measurement used (e.g. parent-report, self-report) leads to different patterns of results (e.g. group differences in emotional self-awareness are present for parent-report only; Huggins et al., 2021).

Although some associations between observer-rated autism behaviours (as measured by the ADOS [Autism Diagnostic Observation Schedule]) are only seen for parent, not self-report, measures of alexithymia (Hobson et al., 2020), this does not discount the potential for measurement error in parent-rated instruments. For example, parents may struggle to report on more internally located aspects of alexithymia, such as how children experience their emotions, leading to underestimation of alexithymia (as suggested by Lampi et al., 2021). However, parent-rated scores on the CAM measure (used in the current study) are associated with predicted manifestations of alexithymia (e.g. diminished facial expression production; Trevisan et al., 2016), suggesting a degree of construct validity. One option for future studies is to use measurements that do not rely on self or parent report to capture alexithymia, for example, degree of difference between self-reported affective states and physiological response (e.g. Gaigg et al., 2018), or number of emotions identified to be experienced by the self across a number of imagined scenarios (Rieffe et al., 2007). A review of research examining differences in alexithymia in autistic populations concluded that studies have largely utilised self-report measures, identifying only three behavioural indices of alexithymia (Huggins et al., 2020), highlighting the importance of developing more objective measures. Multi-modal measurement, including behavioural indices, would allow examination of the type of biases associated with self versus parent-report (e.g. do differences in self vs parent report arise from under-reporting by the self or from over-reporting by the parent), and better understanding of the factors that are associated with alexithymia compared with those driven by other characteristics, including those of the reporter.

Another explanation for the discrepancy in results between previous and current work is cohort effects, such that studies that recruited autistic adults may include more participants who received a diagnosis later in life, compared to the current childhood sample, where everyone received a diagnosis in childhood. This would mean different types of autistic people being included in adult versus adolescent/child samples, who could potentially have different presentations and cognitive profiles.

One final possibility for our null results is lack of variability in alexithymia in the non-autistic group, such that only those with high autistic traits had high alexithymia. This would make it difficult to disentangle their relative contributions. While this could theoretically have contributed to results, the non-significant group × alexithymia interaction term for emotion recognition performance would suggest that the associations between alexithymia and emotion recognition were comparable in autistic groups and non-autistic groups, despite shifted distributions.

In models testing the contribution of autistic traits and alexithymia to ToM abilities, in the whole sample there was an association between autistic traits and ToM (in line with recent meta-analyses; Wilson, 2021), which was most robust (e.g. still significant in secondary covariation models) for the ToM accuracy score – indexing the ability to use mental state understanding to accurately interpret social scenarios. The fact that the association between autistic traits and ToM intentionality – indexing level of mental state descriptions used when interpreting the social scenarios – was non-significant in secondary models and a clear drop in standardised coefficients was noted (β = −0.21 vs β = −0.09) suggests VIQ may explain at least part of the association between autistic traits and this particular metric of ToM. There was no association between alexithymia and ToM intentionality or accuracy. However, analyses testing whether the nature of the alexithymia-social cognition associations differed between non-autistic and autistic youth suggested that the directionality of the association between alexithymia and ToM depended on diagnostic status. In the non-autistic group, higher levels of alexithymia were associated with lower ToM accuracy scores. This result is in line with previous studies of alexithymia in neurotypical adult populations that used the same ToM task (Moriguchi et al., 2006), and reports from large general population samples that find both autistic traits and alexithymia are both unique predictors of self-reported cognitive empathy (Shah et al., 2019). This finding suggests that at least in neurotypical individuals, alexithymia may impact not only the affective domains of empathic understanding (e.g. affective resonance) but also the cognitive aspects of empathy. However, it is still possible that despite the task having less overlap with affective type abilities compared with other ToM tasks (Oakley et al., 2016), individual differences in emotional understanding still influenced performance. We also highlight that associations between alexithymia and ToM became non-significant in secondary models adjusting for parental education, household environment and VIQ, similar to that reported in other covariation analyses (Gökçen et al., 2016). In line with a recent systematic review and meta-analysis (Pisani et al., 2021), we suggest that future work should use ToM tasks that are less dependent on verbal ability (e.g. Penny Hiding task; San José Cáceres et al., 2014). As it stands, our results do not support the suggestion that alexithymia is associated with difficulties in ToM.

In contrast, in the autistic group, there was a positive association between alexithymia and ToM accuracy, although this did not reach statistical significance (p = 0.08). As the moderation by diagnosis analyses were not pre-registered, any conclusions drawn must be treated with caution. However, we note that a differential impact of alexithymia in typically developing compared with autistic youth has been reported elsewhere (Lombardo et al., 2007). One interpretation is that this positive association represents a compensatory strategy in autistic youth with high levels of alexithymia who struggle to automatically identify and understand emotional cues – these challenges may have pushed them to take a ‘cognitive route’ to understanding social situations and cues. This would mean greater reliance on facets of cognitive empathy such as ToM. However, these interesting results require further replication before any conclusions are drawn. At the very least, they highlight the importance of testing the assumption that alexithymia functions in a similar manner in neurotypical individuals compared with autistic individuals.

Key strengths of this work include a moderately sized sample of well-characterised youth with varying levels of autistic traits, pre-registered analyses testing a specific theoretical model and use of well-validated objective cognitive tasks. Current analyses build on our previous work testing the association between autistic traits, callous-unemotional traits and emotion recognition in the same sample (Carter Leno et al., 2022), in terms of delineating what is specific to autism, and what can be explained by often co-occurring traits. One potential limitation is that participants in our autistic group did not complete diagnostic assessments as part of this study; however, we asked very specific questions about the nature of the child’s diagnosis (e.g. when it was obtained, who gave the diagnosis), and our sensitivity analyses excluding participants from the autistic group with lower SCQ scores showed a similar pattern of results. In addition, as our analyses largely focused on autistic traits, we feel that diagnostic misclassification would not substantially change the nature of our results. Finally, as discussed above, the use of parent-report could have led to underestimation of alexithymia, especially in those with less easily observable manifestations of alexithymia, and parents’ own difficulties in emotional literacy (which may likely be more prevalent in parents of autistic children) could have impacted their ability to rate their child’s understanding of emotions. We also acknowledge that the present sample should not be considered fully representative, as indicated by the high VIQ scores of adolescents and education level of participants’ parents. Future work with more diverse populations is required, especially given reports of an association between alexithymia and verbal abilities (Milosavljevic et al., 2016). Finally, we used online versions of cognitive tasks, which could theoretically change the nature of data collected. However, we note that studies which compare cognitive task performance between samples who complete tasks online as compared to in-person find no differences in data quality between settings (Germine et al., 2012), and other studies which collect online and in-person data on a battery of neuropsychological tasks from the same person, 1 week apart, demonstrate the comparability of performance indices (e.g. errors, trials completed and response sensitivity; Backx et al., 2020). The study by Backx and colleagues included the same emotion recognition task we used in the current analyses. They found a strong correlation between median correct RT for online and in-person versions of task (r = 0.73). Studies that have compared task performance between online and in-person performance on the current ToM measure (Frith-Happe Animations) find no differences in ToM condition scores between the two contexts (Livingston et al., 2021). It is, however, worth noting that all the above studies utilised adult samples – to our knowledge no sample has explicitly compared online versus in-person cognitive task performance in children and adolescents. This is an important goal for future research.

In summary, we found autistic traits were significantly associated with lower emotion recognition and ToM ability, and these associations were not significantly changed when adjusting for alexithymia. However, we found evidence that the association between alexithymia and ToM was moderated by diagnostic status, such that negative associations were only found in the non-autistic group. The current results bring into question the applicability of the alexithymia hypothesis to autistic adolescents; however, future research with more robust measures of alexithymia is needed to better understand whether the alexithymia hypothesis is relevant in the adolescent period.

## Supplemental Material

sj-docx-1-aut-10.1177_13623613231221928 – Supplemental material for No association between alexithymia and emotion recognition or theory of mind in a sample of adolescents enhanced for autistic traits Supplemental material, sj-docx-1-aut-10.1177_13623613231221928 for No association between alexithymia and emotion recognition or theory of mind in a sample of adolescents enhanced for autistic traits by Georgianna Moraitopoulou, Hannah Pickard, Emily Simonoff, Andrew Pickles, Rachael Bedford and Virginia Carter Leno in Autism
