# Supplementary material for: No association between alexithymia and emotion recognition or theory of mind in a sample of adolescents enhanced for autistic traits
Source: Autism. 2024 Jan 19;28(8):2066–79. doi: 10.1177/13623613231221928 (PMC11301953; doi:10.1177/13623613231221928)
Supplement: sj-docx-1-aut-10.1177_13623613231221928 – Supplemental material for No association between alexithymia and emotion recognition or theory of mind in a sample of adolescents enhanced for autistic traits [file sj-docx-1-aut-10.1177_13623613231221928.docx]

**No association between alexithymia and emotion recognition or theory of mind in a sample of adolescents enhanced for autistic traits**

**Supplementary Table 1. Diagnostic Information for Autistic Sample (n=75)**

| Diagnostic Label  N (%) | ‘Autism’, ‘Autism Spectrum Disorder’, ‘Autism Spectrum Condition’  ‘Asperger’s’, ‘Asperger’s Syndrome’, ‘High Functioning Autism’  Labels including ‘Pathological Demand Avoidance’  Other | 51 (68%)  18 (24%)  4 (5%)  2 (3%) |
| --- | --- | --- |
| Diagnosis Given By  N (%) | GP/Family Dr  Paediatrician  Psychiatrist  Educational/Clinical Psychologist  Speech and Language Therapist  Other | 3 (4%)  32 (23%)  5 (7%)  21 (28%)  1 (1%)  13 (17%) |
| Age of Diagnosis  Mean (SD; range) | - | 8.28 (3.39; 2 – 15) |

**Supplementary Table 2. Correlations between Key Study Variables**

|  | **1** | **2** | **3** | **4** | **5** | **6** | **7** | **8** | **9** | **10** |
| --- | --- | --- | --- | --- | --- | --- | --- | --- | --- | --- |
| **Sex (1)** | - |  |  |  |  |  |  |  |  |  |
| **Age (years) (2)** | 0.14 |  |  |  |  |  |  |  |  |  |
| **Autism diagnosis (3)** | -0.25*^T^ | 0.02 |  |  |  |  |  |  |  |  |
| **Parental education (4)** | -0.00^T^ | 0.01^T^ | 0.15^T^ |  |  |  |  |  |  |  |
| **CHAOS total (5)** | 0.03 | 0.01 | 0.13 | -0.05 |  |  |  |  |  |  |
| **VIQ (6)** | 0.11 | -0.07 | -0.19** | -0.01 | -0.10 |  |  |  |  |  |
| **AQ total (7)** | -0.17* | 0.14 | 0.65** | -0.19* | 0.26** | -0.26** |  |  |  |  |
| **CAM-PR total (8)** | -0.08 | 0.14 | 0.39** | -0.14 | 0.37* | -0.19* | 0.67** |  |  |  |
| **Emotion recognition (9)** | 0.20* | -0.01 | -0.26** | -0.13 | -0.08 | 0.14 | -0.18* | -0.14 |  |  |
| **ToM Intentionality (10)** | 0.19* | 0.25** | -0.19* | 0.10 | -0.12 | 0.30** | -0.16* | -0.07 | 0.14 |  |
| **ToM Accuracy (11)** | 0.17* | 0.25** | -0.20* | 0.11 | -0.07 | 0.21* | -0.19* | -0.07 | 0.20* | 0.74** |

*p < .05, **p < .01. ^T^ denotes tetrachoric correlations between binary variables.

AQ = Autism Quotient – Adolescent version, CAM-PR = Children’s Alexithymia Measure – parent report, CHAOS = Confusion, Hubbub, and Order Scale, SES = Socio-Economic Status, SCQ = Social Communication Questionnaire, ToM = theory of mind, VIQ = verbal IQ.

**Supplementary Table 3. Secondary Models Testing Associations between Autistic Traits, Alexithymia and Emotion Recognition Task Performance Adjusting for Parental Education, Household CHAOS Total and Verbal IQ (VIQ)**

| **Predictor** | **b** | **β** | **95% CIs** | | **p value** |
| --- | --- | --- | --- | --- | --- |
|  |  |  | **Lower Bound** | **Upper Bound** |  |
| **Age (years)** | 0.05 | 0.01 | -1.12 | 1.22 | 0.93 |
| **Sex** | 3.78 | 0.16 | 0.19 | 7.38 | **0.04** |
| **Autistic Traits** | -0.59 | -0.15 | -1.26 | 0.07 | 0.08 |
| **Parental education** | -4.44 | -0.14 | -8.54 | -0.35 | **0.03** |
| **CHAOS total** | -0.16 | -0.05 | -0.65 | 0.34 | 0.54 |
| **VIQ** | 0.08 | 0.12 | -0.02 | 0.19 | 0.13 |
| **Age (years)** | 0.01 | -0.01 | -1.18 | 1.21 | 0.98 |
| **Sex** | 3.95 | 0.16 | 0.37 | 7.54 | **0.03** |
| **Autistic Traits** | -0.71 | -0.18 | -1.59 | 0.17 | 0.11 |
| **Alexithymia** | 0.04 | 0.04 | -0.17 | 0.25 | 0.72 |
| **Parental education** | -4.33 | -0.14 | -8.49 | -0.18 | **0.04** |
| **CHAOS total** | -0.23 | -0.07 | -0.75 | 0.30 | 0.39 |
| **VIQ** | 0.06 | 0.10 | -0.04 | 0.17 | 0.24 |

CHAOS = Confusion, Hubbub, and Order Scale, VIQ = verbal IQ.

**Supplementary Table 4. Primary Models Testing Associations between Autistic Traits, Alexithymia and ToM Task Performance Adjusting for Goal-Directed (GD) Scores**

|  | **b** | **β** | **95% CIs** |  | **p value** |
| --- | --- | --- | --- | --- | --- |
|  |  |  | **Lower Bound** | **Upper Bound** |  |
| **ToM Intentionality** | | | | | |
| **Age (years)** | 0.14 | 0.28 | 0.07 | 0.21 | **<0.01** |
| **Sex** | 0.22 | 0.12 | -0.05 | 0.49 | 0.11 |
| **GD Intentionality** | 0.14 | 0.11 | 0.02 | 0.26 | **0.03** |
| **Autistic Traits** | -0.06 | -0.20 | -0.10 | -0.02 | **<.01** |
| **Age (years)** | 0.14 | 0.26 | 0.07 | 0.21 | **<0.01** |
| **Sex** | 0.22 | 0.12 | -0.05 | 0.48 | 0.11 |
| **GD Intentionality** | 0.14 | .12 | 0.02 | 0.27 | **0.03** |
| **Autistic Traits** | -0.07 | -0.21 | -0.13 | -0.01 | **0.02** |
| **Alexithymia** | <0.01 | 0.05 | -0.01 | 0.02 | 0.64 |
| **ToM Accuracy** | | | | | |
| **Age (years)** | 0.08 | 0.27 | 0.04 | 0.12 | **<0.01** |
| **Sex** | 0.09 | 0.09 | -0.05 | 0.23 | 0.23 |
| **GD Accuracy** | 0.02 | 0.02 | -0.05 | 0.08 | 0.61 |
| **Autistic Traits** | -0.04 | -0.22 | -0.06 | -0.01 | **<0.01** |
| **Age (years)** | 0.08 | 0.27 | 0.04 | 0.12 | **<0.01** |
| **Sex** | 0.09 | 0.09 | -0.06 | 0.23 | 0.24 |
| **GD Accuracy** | 0.02 | 0.02 | -0.05 | 0.08 | 0.63 |
| **Autistic Traits** | -0.05 | -0.28 | -0.08 | -0.02 | **<0.01** |
| **Alexithymia** | <0.01 | 0.09 | -0.01 | 0.01 | 0.35 |

CHAOS = Confusion, Hubbub, and Order Scale, GD = Goal Directed, ToM = Theory of Mind, VIQ = verbal IQ.

**Supplementary Table 5. Secondary Models Testing Associations between Autistic Traits, Alexithymia and ToM Task Performance Adjusting for Parental Education, Household CHAOS Total and Verbal IQ (VIQ)**

|  | **b** | **β** | **95% CIs** |  | **p value** |
| --- | --- | --- | --- | --- | --- |
|  |  |  | **Lower Bound** | **Upper Bound** |  |
| **ToM Intentionality** | | | | | |
| **Age (years)** | 0.12 | 0.24 | 0.05 | 0.19 | **<0.01** |
| **Sex** | 0.18 | 0.10 | -0.08 | 0.44 | 0.17 |
| **Autistic Traits** | -0.03 | -0.09 | -0.07 | 0.02 | 0.26 |
| **Parental education** | 0.16 | 0.07 | -0.13 | 0.45 | 0.28 |
| **CHAOS total** | -0.01 | -0.06 | -0.05 | 0.02 | 0.44 |
| **VIQ** | 0.01 | 0.28 | 0.01 | 0.02 | **<0.01** |
| **Age (years)** | 0.12 | 0.24 | 0.05 | 0.19 | **<0.01** |
| **Sex** | 0.19 | 0.10 | -0.08 | 0.45 | 0.16 |
| **Autistic Traits** | -0.04 | -0.13 | -0.10 | 0.02 | 0.18 |
| **Alexithymia** | 0.01 | 0.08 | -0.01 | 0.02 | 0.42 |
| **Parental education** | 0.16 | 0.07 | -0.12 | 0.45 | 0.26 |
| **CHAOS total** | -0.02 | -0.09 | -0.06 | 0.02 | 0.31 |
| **VIQ** | 0.01 | 0.28 | 0.01 | 0.02 | **<0.01** |
| **ToM Accuracy** | | | | | |
| **Age (years)** | 0.07 | 0.25 | 0.03 | 0.11 | **<0.01** |
| **Sex** | 0.09 | 0.09 | -0.05 | 0.23 | 0.19 |
| **Autistic Traits** | -0.03 | -0.16 | -0.05 | -0.01 | **0.03** |
| **Parental education** | 0.09 | 0.07 | -0.07 | 0.25 | 0.29 |
| **CHAOS total** | <0.01 | -0.03 | -0.02 | 0.02 | 0.72 |
| **VIQ** | <0.01 | 0.17 | <0.01 | 0.01 | **0.03** |
| **Age (years)** | 0.07 | 0.25 | 0.03 | 0.11 | **<0.01** |
| **Sex** | 0.10 | 0.10 | -0.04 | 0.24 | 0.17 |
| **Autistic Traits** | -0.04 | -0.23 | -0.07 | -0.01 | **0.02** |
| **Alexithymia** | 0.01 | 0.12 | -0.01 | 0.01 | 0.20 |
| **Parental education** | 0.09 | 0.07 | -0.07 | 0.25 | 0.27 |
| **CHAOS total** | -0.01 | -0.06 | -0.03 | 0.01 | 0.44 |
| **VIQ** | 0.01 | 0.17 | 0.01 | 0.01 | **0.03** |

CHAOS = Confusion, Hubbub, and Order Scale, ToM = Theory of Mind, VIQ = verbal IQ.

**Supplementary Table 6. Models Testing Associations between Autistic Traits, Alexithymia and Emotion Recognition Task Performance Excluding Autistic Participants with SCQ < 15**

| **Predictor** | **b** | **β** | **95% CIs** | | **p value** |
| --- | --- | --- | --- | --- | --- |
|  |  |  | **Lower Bound** | **Upper Bound** |  |
| **Age (years)** | 0.07 | 0.01 | -1.12 | 1.25 | 0.91 |
| **Sex** | 3.36 | 0.14 | -0.43 | 7.15 | 0.08 |
| **Autistic traits** | -0.82 | -0.20 | -1.47 | -0.17 | **0.01** |
| **Age (years)** | 0.07 | 0.01 | -1.12 | 1.26 | 0.91 |
| **Sex** | 3.36 | 0.14 | -0.44 | 7.16 | 0.08 |
| **Autistic traits** | -0.88 | -0.22 | -1.70 | -0.07 | **0.03** |
| **Alexithymia** | 0.03 | 0.02 | -0.17 | 0.22 | 0.79 |

**Supplementary Table 7. Models Testing Associations between Autistic Traits, Alexithymia and ToM Task Performance Excluding Autistic Participants with SCQ <15**

|  | **b** | **β** | **95% CIs** |  | **p value** |
| --- | --- | --- | --- | --- | --- |
|  |  |  | **Lower Bound** | **Upper Bound** |  |
| **ToM Intentionality** | | | | | |
| **Age (years)** | 0.12 | 0.24 | 0.05 | 0.19 | **<0.01** |
| **Sex** | 0.25 | 0.14 | -0.03 | 0.53 | 0.08 |
| **Autistic traits** | -0.04 | -0.15 | -0.09 | -0.01 | 0.05 |
| **Age (years)** | 0.12 | 0.24 | 0.05 | 0.19 | **<0.01** |
| **Sex** | 0.25 | 0.14 | -0.03 | 0.53 | 0.08 |
| **Autistic traits** | -0.05 | -0.18 | -0.12 | 0.01 | **0.09** |
| **Alexithymia** | <0.01 | 0.05 | -0.01 | 0.02 | 0.59 |
| **ToM Accuracy** | | | | | |
| **Age (years)** | 0.07 | 0.24 | 0.02 | 0.11 | **<0.01** |
| **Sex** | 0.11 | 0.11 | -0.04 | 0.26 | 0.17 |
| **Autistic traits** | -0.03 | -0.19 | -0.05 | -0.01 | **0.01** |
| **Age (years)** | 0.07 | 0.24 | 0.02 | 0.11 | **<0.01** |
| **Sex** | 0.11 | 0.11 | -0.05 | 0.26 | 0.17 |
| **Autistic traits** | -0.04 | -0.23 | -0.07 | -0.01 | **0.02** |
| **Alexithymia** | <0.01 | 0.07 | -0.01 | 0.01 | 0.76 |

**Supplementary Table 8. Models Testing Associations between Autistic Traits, Alexithymia and Emotion Recognition Task Performance Adjusting for Parental Education, Household CHAOS Total and Verbal IQ (VIQ) Excluding Autistic Participants with SCQ <15**

| **Predictor** | **b** | **β** | **95% CIs** | | **p value** |
| --- | --- | --- | --- | --- | --- |
|  |  |  | **Lower Bound** | **Upper Bound** |  |
| **Age (years)** | -0.14 | -0.02 | -1.41 | 1.12 | 0.82 |
| **Sex** | 3.12 | 0.13 | -0.78 | 7.01 | 0.12 |
| **Autistic Traits** | -0.75 | -0.19 | -1.45 | -0.05 | **0.04** |
| **Parental education** | -4.55 | -0.15 | -8.80 | -0.31 | **0.04** |
| **CHAOS total** | -0.20 | -0.06 | -0.72 | 0.32 | 0.44 |
| **VIQ** | 0.06 | 0.09 | -0.05 | 0.16 | 0.30 |
| **Age (years)** | -0.13 | -0.02 | -1.41 | 1.16 | 0.85 |
| **Sex** | 3.19 | 0.13 | -0.68 | 7.06 | 0.11 |
| **Autistic Traits** | -0.90 | -0.22 | -1.82 | 0.02 | 0.06 |
| **Alexithymia** | 0.07 | 0.06 | -0.16 | 0.29 | 0.57 |
| **Parental education** | -4.52 | -0.15 | -8.79 | -0.26 | **0.04** |
| **CHAOS total** | -0.25 | -0.08 | -0.78 | 0.29 | 0.36 |
| **VIQ** | 0.05 | 0.09 | -0.05 | 0.16 | 0.33 |

CHAOS = Confusion, Hubbub, and Order Scale, VIQ = verbal IQ.

**Supplementary Table 9. Models Testing Associations between Autistic Traits, Alexithymia and ToM Task Performance Adjusting for Parental Education, Household CHAOS Total and Verbal IQ (VIQ) Excluding Autistic Participants with SCQ <15**

|  | **b** | **β** | **95% CIs** |  | **p value** |
| --- | --- | --- | --- | --- | --- |
|  |  |  | **Lower Bound** | **Upper Bound** |  |
| **ToM Intentionality** | | | | | |
| **Age (years)** | 0.03 | 0.06 | -0.05 | 0.11 | 0.45 |
| **Sex** | 0.21 | 0.11 | -0.07 | 0.48 | 0.14 |
| **Autistic Traits** | -0.01 | -0.03 | -0.06 | 0.04 | 0.71 |
| **Parental education** | 0.23 | 0.10 | -0.06 | 0.52 | 0.12 |
| **CHAOS total** | -0.02 | -0.08 | -0.06 | 0.02 | 0.31 |
| **VIQ** | 0.02 | 0.32 | 0.01 | 0.03 | **<0.01** |
| **Age (years)** | 0.03 | 0.06 | -0.05 | 0.11 | 0.44 |
| **Sex** | 0.21 | 0.12 | -0.06 | 0.48 | 0.13 |
| **Autistic Traits** | -0.02 | -0.07 | -0.08 | 0.04 | 0.50 |
| **Alexithymia** | 0.01 | 0.06 | -0.01 | 0.02 | 0.53 |
| **Parental education** | 0.23 | 0.10 | -0.05 | 0.52 | 0.11 |
| **CHAOS total** | -0.02 | -0.10 | -0.06 | 0.01 | 0.22 |
| **VIQ** | 0.02 | 0.32 | 0.01 | 0.02 | **<0.01** |
| **ToM Accuracy** | | | | | |
| **Age (years)** | 0.04 | 0.13 | -0.01 | 0.08 | 0.13 |
| **Sex** | 0.11 | 0.10 | -0.05 | 0.26 | 0.17 |
| **Autistic Traits** | -0.02 | -0.11 | -0.04 | 0.01 | 0.16 |
| **Parental education** | 0.12 | 0.09 | -0.05 | 0.28 | 0.17 |
| **CHAOS total** | -0.01 | -0.04 | -0.03 | 0.01 | 0.57 |
| **VIQ** | 0.01 | 0.19 | 0.00 | 0.01 | **0.03** |
| **Age (years)** | 0.04 | 0.13 | -0.01 | 0.08 | 0.13 |
| **Sex** | 0.11 | 0.11 | -0.04 | 0.26 | 0.16 |
| **Autistic Traits** | -0.03 | -0.17 | -0.06 | 0.01 | 0.10 |
| **Alexithymia** | 0.00 | 0.10 | 0.00 | 0.01 | 0.33 |
| **Parental education** | 0.12 | 0.09 | -0.05 | 0.28 | 0.16 |
| **CHAOS total** | -0.01 | -0.07 | -0.03 | 0.01 | 0.38 |
| **VIQ** | 0.01 | 0.19 | 0.00 | 0.01 | **0.03** |

CHAOS = Confusion, Hubbub, and Order Scale, ToM = Theory of Mind, VIQ = verbal IQ.
